# Partial Restoration of Macrophage Alteration from Diet-Induced Obesity in Response to *Porphyromonas gingivalis* Infection

**DOI:** 10.1371/journal.pone.0070320

**Published:** 2013-07-29

**Authors:** Guilhem Richard, Niraj Trivedi, Calin Belta, Salomon Amar

**Affiliations:** 1 Center for Anti-Inflammatory Therapeutics, Boston University Goldman School of Dental Medicine, Boston, Massachusetts, United States of America; 2 Department of Bioinformatics, Boston University, Boston, Massachusetts, United States of America; University of Florida, College of Dentistry & The Emerging Pathogens Institute, United States of America

## Abstract

Obesity is a chronic inflammatory disease that weakens macrophage innate immune response to infections. Since M1 polarization is crucial during acute infectious diseases, we hypothesized that diet-induced obesity inhibits M1 polarization of macrophages in the response to bacterial infections. Bone marrow macrophages (BMMΦ) from lean and obese mice were exposed to live *Porphyromonas gingivalis* (*P. gingivalis*) for three incubation times (1 h, 4 h and 24 h). Flow cytometry analysis revealed that the M1 polarization was inhibited after *P. gingivalis* exposure in BMMΦ from obese mice when compared with BMMΦ from lean counterparts. Using a computational approach in conjunction with microarray data, we identified switching genes that may differentially control the behavior of response pathways in macrophages from lean and obese mice. The two most prominent switching genes were thrombospondin 1 and arginase 1. Protein expression levels of both genes were higher in obese BMMΦ than in lean BMMΦ after exposure to *P. gingivalis*. Inhibition of either thrombospondin 1 or arginase 1 by specific inhibitors recovered the M1 polarization of BMMΦ from obese mice after *P. gingivalis* exposure. These data indicate that thrombospondin 1 and arginase 1 are important bacterial response genes, whose regulation is altered in macrophages from obese mice.

## Introduction

Previous works showed that the macrophage, a cell responsible for the secretion of inflammatory mediators [1], [2], [3], tends to accumulate in adipose tissue of obese subjects as compared with lean subjects [4]. However, rather than having a heightened response to infection due to elevated amounts of inflammatory signals and immune cells, previous studies found that the response to infection in obese subjects was instead impaired [5], [6], [7].

Smith *et al.* [5] showed that obese mice had a 6.6-fold higher mortality rate than lean mice in response to infection. The obese mice mounted an immune response that attempted to recover from the infection, as indicated by levels of interleukin (IL)-6 and TNF. These cytokines, however, required 6 days to reach circulating concentrations expected after only 3 days in lean mice [5]. Mice with diet-induced obesity exposed to oral infection or systemic inoculation of live *Porphyromonas gingivalis* (*P. gingivalis*) developed a blunted inflammatory response with reduced expression of TNF, IL-6, and serum amyloid protein A (SAA) when compared with lean mice [7]. A major challenge is to determine the factors that are responsible for the delay in the response to infection in obese subjects. Identifying such factors may have therapeutic implications for obese humans fighting infections.

Macrophage phenotypes can be characterized as classical (M1) or alternative (M2) activation states. Proinflammatory cytokines, such as interferon (IFN)-γ and interleukin (IL)-1β, and inducers of TNF, such as lipopolysaccharide (LPS), promote M1 macrophage differentiation [8]. M1 macrophages produce proinflammatory cytokines, such as TNF, IL-6, and IL-12, and are linked to increased immune response and tissue destruction [9]. IL-12 is a powerful signal for the generation of T helper type 1 lymphocyte (T_H_1) responses required to eliminate intracellular pathogens [10]. In contrast, M2 macrophages, which are induced in response to IL-4 and IL-13, dampen the inflammatory process by producing anti-inflammatory factors, such as IL-10 and TGF-β, and are associated with angiogenesis and tissue repair [8], [11]. Obesity induces M1 polarization in adipose tissue macrophages [9]. However, its role on BMMΦ polarization is unclear.

A widespread approach to quantify the differences between two experimental conditions is to identify and rank the genes that are differentially expressed between the two conditions [12], [13]. However, identifying the genes that cause the phenotypic differences is difficult: due to gene network interactions, the causative genes may not be the most differentially expressed. Ideally, a detailed and accurate mathematical model of relevant gene interactions could help in answering this question [14], [15]. The construction of such a model, which includes topological reconstruction and kinetic parameters estimation, is currently an impossible task at the genome scale. Therefore, we propose a simple, computationally efficient method that combines machine learning with database searches to predict such genes from a small amount of gene expression data.

In the present study, we hypothesized that diet-induced obesity exerted novel effects on M1/M2 polarization of macrophages in response to *P. gingivalis* infection. To this aim, we evaluated the effects of diet-induced obesity on BMMΦ differentiation and polarization. Using cDNA arrays and bioinformatics techniques, we identified switching genes that have critical effects on M1/M2 polarization of macrophages from obese mice after *P. gingivalis* infection.

## Materials and Methods

### Bacteria culture

*P. gingivalis* 381 (ATCC, Manassas, VA) was grown in brain-heart infusion broth (Gibco) enriched with hemin (5 µg/ml) and menadione (100 ng/ml) in an anaerobic atmosphere (85% N2, 10% H2, 5% CO2) for 24 to 48 hours at 37°C. Cells were grown until the culture reached an optical density of 0.8 at 560 nm. After centrifugation, the cell pellet was resuspended in 1 mL bacteriostatic 0.9% sodium chloride. Cell concentration was measured by absorbance at 560 nm.

### Macrophage culture and in vitro infection

For 16 weeks, C57BL/6J mice were fed a high fat diet to induce obesity [7]. Control (lean) mice were maintained for the same period on standard laboratory chow. No diabetic animals were included in the study. Obese and lean mice were euthanized via isofluorane exposure. The femurs and tibulas were dissected from mice and bone marrow cells were flushed from medullar cavity of the bones with PBS and then cultured in 30% L-929 conditioned RPMI media at a density of 10^6^ cells/ml [16]. After one week at 37°C, cells differentiated into bone marrow macrophages (BMMΦ) and cell culture medium was changed with fresh RPMI-1640 media 1 h before the experiments. Cultured *P. gingivalis* were added to the BMMΦ cultures at 20 times the concentration of BMMΦ (MOI = 20∶1) [12]. BMMΦ incubated with *P. gingivalis* for three different lengths of time: 1 h, 4 h, and 24 h. Cells were collected for further studies after each incubation time.

### Flow cytometry

Flow cytometry (FACS) analysis was performed with routine protocols using the FACSCan flow cytometer (BD Immunocytometry Systems), with antibodies against IL-10, IL-12, CD11b and F4/80 (eBioscience). For cytoplasmic staining, cells were treated with Brefeldin A, and were stained and analyzed by using a fixation & permeabilization kit (eBioscience) following the recommended protocols.

### Cytokine Measurement

Concentrations of cytokines in sera or cell culture supernatants were measured by using Bio-Plex Luminex assay [7]. TNF and IL-10 levels were determined using BD Bioscience-PharMingen ELISA kits.

### RNA extraction and cDNA synthesis

Total RNA from macrophages were extracted and purified using an RNA extraction kit from Fujifilm. cDNA synthesis using reverse transcriptase PCR was performed via protocols and reagents from the Bio-Rad iScript cDNA Synthesis kit.

### cDNA microarray

cDNA from BMMΦ of lean and obese mice treated with live *P. gingivalis* for three lengths of time (1, 4, and 24 h) were hybridized on Affymetrix Mouse Genome 430 2.0 Arrays, as described in our previous study [12]. Hybridization was performed on three replicates at each time point (18 arrays total). Normalization of microarray data was performed using Bioconductor; part of the software package R. Quantile normalization was used to reduce the variability from the three replicates at each time point. Loess normalization was employed to reduce system noise between time points. Differentially expressed genes between cells from obese or lean mice were identified using the limma package in R at each time point. The cDNA microarrays were submitted to the Gene Expression Omnibus (GEO) database (accession number: GSE47414, http://www.ncbi.nlm.nih.gov/geo/query/acc.cgi?acc=gSE47414).

### Switching genes and pathway enrichment

We used Self-Organizing Maps (SOMs), a machine learning method developed by Kohonen [17], to capture genes that behave similarly in either lean or obese macrophages in response to infection. A SOM spatially clusters genes that have similar expression levels over time. Genes in a cluster have temporal dynamics that can all be described by the same average behavior. Genes in adjacent clusters have close, but different behaviors. The more the clusters are distant on the SOM, the more these differences become significant. Two separate SOMs were generated from the microarray data of lean and obese mouse macrophages in response to *in vitro* infection, one using the lean dataset and one using the obese dataset. The SOM algorithm was executed using GEDI (Children's Hospital Boston) [18]. We used a 35×36 cluster map and each cluster was represented by the average behavior of its genes. Information about the genes on the Affymetrix array sets, such as gene name, gene abbreviation, Entrez GeneID, etc., were downloaded from EASE [19], loaded into a MySQL database (Oracle Corporation, Redwood City, CA), and accessed by SequelPro (North of Three, Australia).

**Figure 1:**
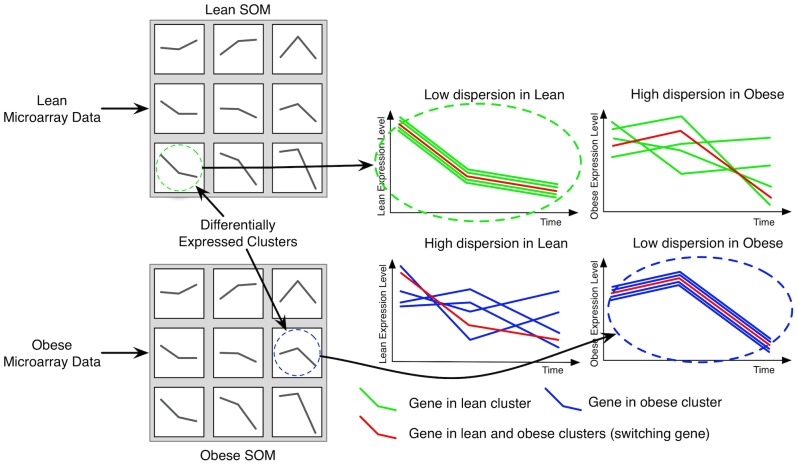
Schematic of the procedure for identifying switching genes. Microarray data for the BMMΦ from lean and obese mice in response to infection was used to create two Self-Organizing Maps (SOMs). Differentially expressed clusters from the lean and obese SOMs with at least one gene in common were identified and plotted as shown. The left and right graphs show the behavior of genes in the lean and obese BMMΦ, respectively. The green dashed circle refers to a lean cluster, as taken from the lean SOM, where all the gene trajectories, shown in green, have similar behavior over the three time points. These genes lose their clustered behavior over time when expressed in obese BMMΦ. Similarly, the blue dashed circle refers to an obese cluster, as taken from the obese SOM. All the trajectories shown in blue are clustered when genes are expressed in obese BMMΦ, and dispersed when genes are expressed in lean BMMΦ. The red line represents the time behavior of the switching gene, which moves from the lean cluster to the obese cluster due to the change in condition. This gene appears to activate/deactivate the genes in the lean and obese clusters.

We define a “switching” gene as a gene that is present in differentially expressed clusters between the lean SOM and the obese SOM, and which satisfies the following three additional properties (see Figure 1):

1. The sets of genes that cluster with the switching gene in the lean and obese SOM are disjoint; 2. All the genes that cluster with the switching gene in the lean (obese) SOM are not part of the same cluster in the obese (lean) SOM: gene expressions appear clustered in the lean (obese) dataset, and dispersed in the obese (lean) dataset; 3. The sets of genes in either the lean or obese clusters enrich known pathways. Based on this definition, a switching gene has a potentially “pivotal” behavior due to its property of activating/deactivating biologically relevant pathways. Such a gene is likely to cause on/off switches from a function to another in the lean vs. the obese macrophages.

We assess the dispersion of clustered genes by summing the standard deviation of all gene expressions at each time point. We computed the Euclidean distances (*i.e.*, the square roots of the sums of the squares of the differences at each time point) between the average behaviors of all pairs of clusters for both lean and obese SOMs using MATLAB (The Mathworks, Natick, MA) to characterize the difference of expression between genes from different clusters. We searched for switching genes with MySQL and ranked the corresponding pairs of lean and obese clusters according to their Euclidean distance (*i.e.*, differential expression).

### Pathway Enrichment

The software package DAVID [20] was used to determine pathway enrichment in the lean and obese clusters of each switching gene. A pathway was considered enriched by a cluster if at least two genes in the cluster belonged to a pathway and the EASE score, a modified Fisher Exact Test from DAVID, was <0.1. If no pathway was enriched, the spatial map of the SOM was used to capture more genes in the cluster. Since clusters adjacent to each other have similar behaviors, genes in clusters immediately adjacent to the original cluster were included only if the genes had a distance less than one standard deviation from their switching gene. The resulting genes in the cluster were capped to 250 genes as suggested by DAVID [21].

### Quantitative RT-PCR

Primer-BLAST [22] was used to select primers for genes to be tested as shown in Table S1. Quantitative PCR was performed on all samples using iQSYBR Green Supermix (Bio-Rad). β-actin was used to normalize the data. Quantitative PCR was considered to validate the microarray data if both methods showed the same relative intensity and/or had the same behavioral trend for a given gene.

### Western blot

Western blots were performed using antibodies against thrombospondin 1, arginase 1 or β-actin (Santa Cruz Biotechnology) according to previous protocols [12].

## Results

### Diet-induced obesity inhibits M1 polarization of bone marrow macrophages after *P. gingivalis* infection

**Figure 2:**
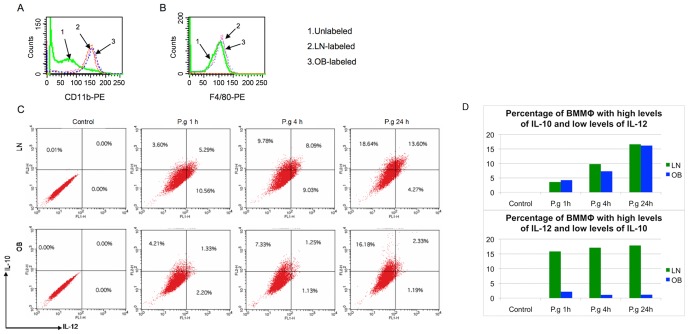
M1 polarization is inhibited in BMMΦ from obese mice after *P.* *gingivalis* infection. (A) Uninfected BMMΦ from lean (LN) and obese (OB) mice were left unlabeled or were labeled with PE-conjugated anti-mouse CD11b and analyzed by FACS to determine macrophages differentiation. (B) Uninfected BMMΦ from LN and OB mice were left unlabeled or were labeled with PE-conjugated anti-mouse F4/80 and analyzed by FACS to determine macrophages maturation. (C) BMMΦ from LN and OB mice were infected with *P. gingivalis* (P.g) for 1, 4 and 24 h. Cells were collected and labeled with PE-conjugated anti-mouse IL-12 and FITC-conjugated anti-mouse IL-10. The percentages of M1 and M2 macrophages were measured by FACS analysis. Data represent one of three independent experiments with consistent results. (D) Percentage of BMMΦ from LN and OB mice that present an M1 polarization (high levels of IL-12 and low levels of IL-10) and an M2 polarization (high levels of IL-10 and low levels of IL-12).

Our previous studies showed that bone marrow macrophages (BMMΦ) from obese mice exhibited blunted proinflammatory responses when challenged with live *P. gingivalis* [6], [7], [23]. In this study, we tested whether diet-induced obesity affects BMMΦ M1/M2 polarization. We cultivated bone marrow cells in the presence of L929 conditioned medium to induce the differentiation of these cells into mature BMMΦ [16]. Flow cytometry (FACS) analysis confirmed that BMMΦ expressed macrophage specific marker CD11b and that there was no difference in its expression level between BMMΦ from lean and obese mice (Figure 2A). This suggests that diet-induced obesity does not affect macrophage differentiation. BMMΦ derived from obese and lean mice were labeled with macrophage maturation marker F4/80 to determine if diet-induced obesity affects the maturation status of BMMΦ. Marker expression level was measured by FACS analysis. Results showed that diet-induced obesity also did not affect the maturation of BMMΦ *in vitro* (Figure 2B). BMMΦ obtained from lean and obese mice were stimulated with live *P. gingivalis* for 1, 4, and 24 h to analyze the M1/M2 polarization of macrophages after infection. Cells were analyzed by FACS for the cytoplastic expression of IL-12 and IL-10, hallmark cytokines of M1 and M2 polarization, respectively (Figure 2C). We summarized the results from this analysis by counting the BMMΦ from lean and obese mice that showed high levels of IL-12 and low levels of IL-10 on one side, and high levels of IL-10 and low levels of IL-12 on the other side (Figure 2D). As shown in Figures 2C and 2D, the M1 polarization of BMMΦ from obese mice was inhibited during *P. gingivalis* infection at all tested time points when compared with BMMΦ from lean mice. The M2 polarization was however comparable between BMMΦ from lean and obese mice.

### Switching genes identified between lean and obese bone marrow macrophages

**Figure 3:**
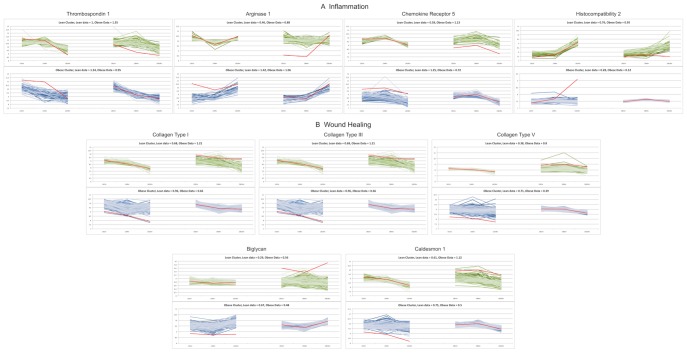
Top nine switching genes. Switching genes were identified using the procedures described in the Materials and Methods. The top 9 switching genes were thrombospondin 1 (Thbs1), arginase (Arg1), chemokine (C-C motif) receptor 5 (Ccr5), histocompatibility complex 2 (H2-Q6), Collagen types I (Col1a2), III (Col3a1), and V (Col5a2), biglycan (Bgn), and caldesmon 1 (Cald1). We divided switching genes into two categories: (A) inflammation and (B) wound healing genes. For each switching gene (shown in red), the top (bottom) plot displays the dynamics of the genes that cluster with the switching gene in the lean (obese) dataset. The left and right parts of the plots show the gene dynamics in BMMΦ from lean and obese mice, respectively. For each condition (lean or obese), we show the dispersion of the dynamics (*i.e.* the sum of the standard deviations of all gene expressions at each time point in the lean and obese dataset) of the genes clustering with the switching gene. In each case, the dynamics of the genes clustering with the switching gene in lean (obese) macrophages are more dispersed in the obese (lean) dataset.

To determine the switching genes that alter the M1 polarization of BMMΦ from obese mice during *P. gingivalis* exposure, we further investigated the gene profiles of BMMΦ infected with *P. gingivalis* for 1, 4, and 24 h. We found a total of 158 genes that were differentially expressed between lean and obese BMMΦ (FDR≤2 and p<0.05). We identified 61 switching genes using our computational approach described in Materials and Methods and illustrated in Figure 1. The most prominent switching genes were thrombospondin 1 (Thbs1), arginase (Arg1), chemokine (C-C motif) receptor 5 (Ccr5), histocompatibility complex 2 (H2-Q6), Collagen types I (Col1a2), III (Col3a1), and V (Col5a2), biglycan (Bgn), and caldesmon 1 (Cald1) (Figure 3). These genes had the largest distance between their lean and obese clusters and also had the highest level of differential expression. We divided the switching genes into two categories based on their functions: inflammation genes (Thbs1, Arg1, Ccr5, and H2-Q6) (Figure 3A) and wound healing genes (Col1a2, Col3a1, Col5a2, Bgn, and Cald1) (Figure 3B).

### Pathway enrichment for switching genes

We used the software package DAVID to find pathways enriched by the 158 differentially expressed genes [20]. All the enriched pathways had an EASE score <0.1 and a minimum of two genes per pathway, as per the default of DAVID. The most prominent enriched pathways were Cytokine-cytokine receptor interaction, Toll-like receptor pathway, RIG-I-like receptor signaling pathway, Chemokine signaling pathway, Cytosolic DNA-sensing pathway, ECM-receptor interaction, Systemic lupus erythematosus, and NOD-like receptor signaling pathway. The behaviors of pathways over time can be inferred by correlating the associations of genes with the enriched pathways (Table S2) and with the cluster time-behaviors (Figure 3). For example, the Toll-like receptor pathway from the lean cluster of Thbs1 (Table S2), when paired with the expression data in Figure 3, showed that the pathway was highly expressed at the first two time points (1 and 4 hours), but by 24 hours, genes in this pathway had decreased their expression level.

### Validation of switching genes

**Figure 4:**
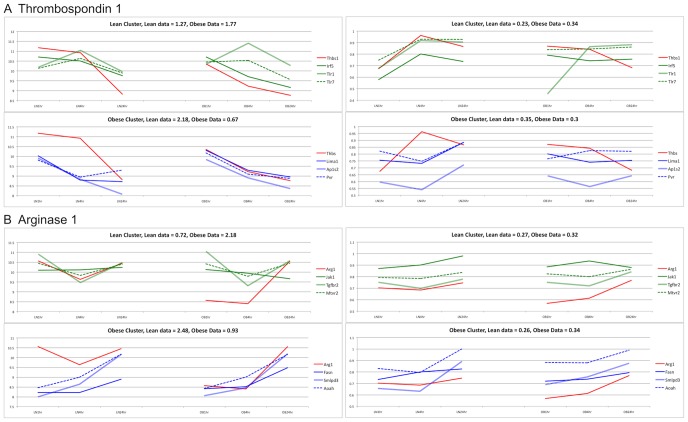
Validation of microarray data by RT-PCR. The mRNA levels of two switching genes, Thbs1 (A) and Arg1 (B), together with a subset of the genes clustering with them, were validated using quantitative RT-PCR. Microarray data are shown on the left, quantitative RT-PCR validation on the right. Results are displayed in the same manner as Figure 3. Switching genes are shown in red. The top (bottom) part of the plots shows the dynamics of the genes clustering with the switching gene in lean (obese) dataset.

We validated the top two switching genes, Thbs1 and Arg1, and a subset of their clusters from lean and obese mice using quantitative PCR, as shown in Figure 4. Note that the genes associated with Thbs1 were found to correlate with the microarray data by intensity and behavior (Figure 4A). Arg1 and the genes within its corresponding cluster, however, did not correlate by expression levels, but correlated by behavior (Figure 4B).

### Thbs1 and Arg1 protein levels are higher in obese bone marrow macrophages than in lean bone marrow macrophages after *P. gingivalis* infection

**Figure 5:**
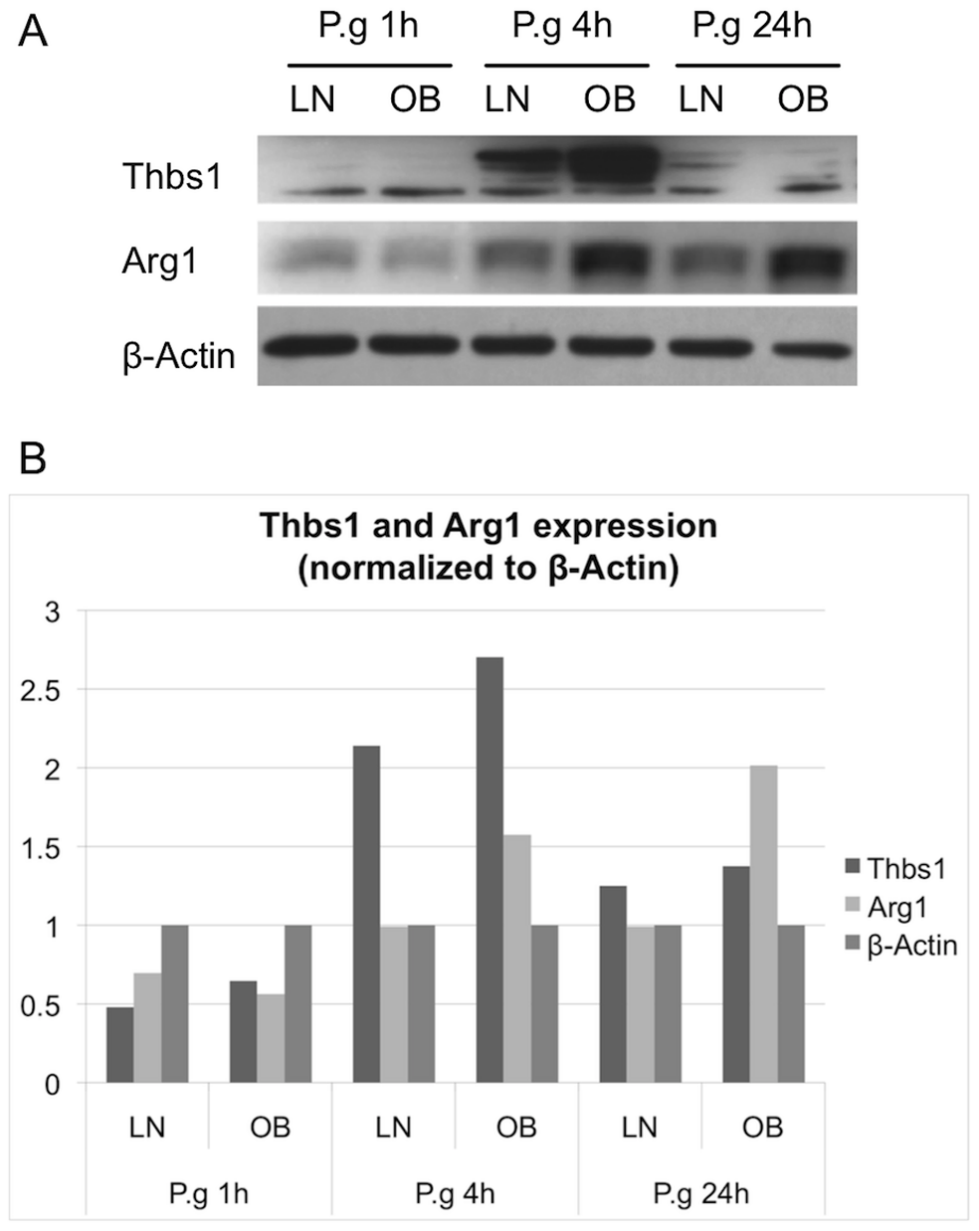
Thbs1 and Arg1 expression after *P.* *gingivalis* infection. BMMΦ from lean (LN) and obese (OB) mice were stimulated with *P. gingivalis* (P.g) for 1, 4 and 24 h. (A) The protein levels of Thbs1 and Arg1 were detected by Western blot using whole cell lysates. (B) The intensities of the bands were converted into quantitative data. The expression of Thbs and Arg1 were normalized to β-Actin.

To further determine the protein expression of Thbs1 and Arg1 after BMMΦ infection with *P. gingivalis*, BMMΦ were generated from lean and obese mice and stimulated with live *P. gingivalis* for 1, 4, and 24 h. Western blot analysis demonstrated that BMMΦ from obese mice produced much more Thbs1 and Arg1 proteins after 4 h infection compared with BMMΦ from lean mice (Figure 5). Arg1 levels were still much higher in BMMΦ from obese mice after 24 h infection. BMMΦ from obese and lean mice produced more comparable amounts of Thbs1 protein after 24 h infection, with a slight overproduction still observed in obese mice. Levels of Thbs1 and Arg1 in uninfected BMMΦ were comparable to the ones in BMMΦ after 1 h infection (data not shown).

### Inhibition of Thbs1 or Arg1 restores M1 polarization of bone marrow macrophages from obese mice

**Figure 6:**
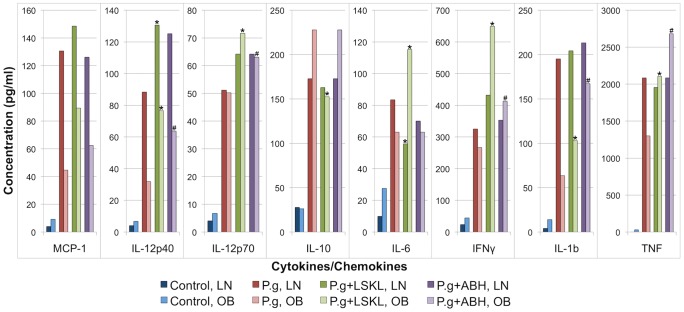
Inhibition of Thbs1 or Arg1 reestablishes normal concentrations of cytokines in BMMΦ from obese mice. BMMΦ from lean (LN) and obese (OB) were treated with Thbs1 inhibitor LSKL, or with Arg1 inhibitor ABH ammonium salt, immediately before the exposure to *P. gingivalis* (P.g). After 4 h incubation, cytokines in the cell culture supernatants were measured by Bio-Plex cytokine array. The data presented are means of 3 replicates. *P<0.05 (P.g vs. P.g+LSKL); #*P*<0.05 (P.g vs. P.g+ABH).

To determine whether the increased expression of Thbs1 or Arg1 in obese BMMΦ after *P. gingivalis* infection is responsible for the inhibition of M1 polarization of BMMΦ from obese mice, we treated the BMMΦ from lean and obese mice with 1 µM [24] of peptide LSKL (Enzo Life Science) and 100 µM [25] of 2(S)-amino-6-boronohexanoic acid (ABH) ammonium salt (AnaSpec,Inc), specific inhibitors for Thbs1 and Arg1, respectively, immediately before the stimulation of BMMΦ with live *P. gingivalis*. In addition, we treated BMMΦ from lean and obese mice with 1 µM of peptide SLLK (AnaSpec, Inc) and 10 mM of D-ornithine (Sigma Aldrich), inert controls for LSKL and ABH ammonium salt, respectively, immediately before the stimulation of BMMΦ with live *P. gingivalis* and measured the production of TNF to rule out any off-target effect. Inhibition of either Thbs1 or Arg1 restored the M1 polarization level of BMMΦ from obese mice to the level of BMMΦ from lean mice. We verified the efficacy of each inhibitor to promote the M1 polarization of macrophages after a *P. gingivalis* infection by following the concentrations of 8 cytokines: MCP-1, IL-12p40, IL-12p70, IL-10, IL-6, INF-γ, IL-1b, and TNF (Figure 6). We performed a Student's *t*-test to evaluate the significance of the results and used Bonferroni correction to adjust for multiple comparisons. Both inhibitors had little to no effect on the production of cytokines in BMMΦ from lean mice. Only the production of IL-12p40 and IL-6 were significantly affected by LSKL. ABH did not significantly change the expression level of any of the tested cytokines. BMMΦ from obese mice were however sensitive to the presence of either LSKL or ABH; in particular pro-inflammatory mediators TNF, INF-γ, IL-6 were found significantly up regulated by LSKL and ABH, and anti-inflammatory mediator IL-10 was found down regulated by LSKL. Treatment with either SLLK or D-ornithine had no effect on TNF concentration. These observations seem to indicate that the inhibition of Thbs1 or Arg1 promotes the M1 polarization of macrophages specifically in obese mice.

## Discussion

Polarized macrophages have been broadly classified into two groups: M1 and M2 macrophages. Activation of the M1 program after bacterial infections is usually considered a protection from acute infectious diseases [26]. The protective role of M1 macrophages has been exemplified in mice deficient for components of the IL-12 pathway [27]. However, excessive or prolonged M1 polarization can lead to tissue injury and contribute to pathogenesis [28]. In contrast, M2 macrophages play a critical role in the resolution of inflammation by producing anti-inflammatory mediators, such as IL-10 and TGF-β [29], [30].

Diet-induced obesity-associated inflammation is characterized by an increased abundance of M1 macrophages in the adipose tissue [9], which are postulated to be major sources of several molecular mediators, such as TNF, IL-6, and C-reactive protein [31]. Somewhat paradoxically, obese subjects present weakened antibacterial immunity rather than having a heightened immune response to infections [5], [6], [7], [23]. Our previous studies have demonstrated that weakened antibacterial immunity, such as attenuated inflammatory reaction of macrophages in response to bacterial challenges, results at least in part from the disruption of the TLR2 signaling pathway of obese individuals [6], [7]. In the present study, we have now further revealed that the M1 polarization of macrophages after bacterial infections appears to be compromised in obese mice (Figure 2). This might be another important reason for the weakened antibacterial immunity of obese animals.

The common response of macrophages after bacterial infections is to up-regulate the expression of a group of genes that are mainly involved in the M1 polarization [28]. We postulated that there may be a switching gene that redirects the phenotypic switch of macrophage polarization in obese macrophages after bacterial infections. We first obtained genes expression levels from bone marrow macrophages (BMMΦ) of lean and obese mice exposed to live *P. gingivalis* during 1, 4, and 24 h using cDNA microarrays. Then, we analyzed gene dynamics with Self Organizing Maps (SOMs) to group genes that presented similar behaviors over time. Studies showed that genes with similar dynamics are more likely to interact with each other and tend to belong to the same pathway [32], [33]. After generating a lean and an obese SOM, we searched for genes that were clustered in one condition (lean or obese) and dispersed in the other one, and that enriched known pathways. Through further analysis, we showed that a single differentially expressed gene, the switching gene, was part of a cluster in both lean macrophages and obese macrophages (Figure 1). The lean and obese clusters had different levels of expression, which suggested that the genes belonging to the two clusters had a low probability of interacting. We therefore hypothesized that the switching gene is a bridge of interactions between these two clusters. By comparing the enriched pathways of the set of differentially expressed genes to the enriched pathways from the lean and obese clusters, we identified thrombospondin 1 (Thbs1) and arginase 1 (Arg1) as the top two switching genes involved in macrophage inflammatory reactions (Figure 3).

Thbs1 is a regulator of inflammation. Activation of TGF-β by Thbs1 contributes to immune homeostasis and immune tolerance [34]. Our microarray data showed that genes clustering with Thbs1 in lean (obese) macrophages tend to have dispersed dynamics in obese (lean) macrophages (Figure 3). Using peptide LSKL, a specific inhibitor for Thbs1, we confirmed that Thbs1 is located in a genetic network such that its protein expression inhibits M1 polarization (Figure 6).

Arg1 has also a role in the inflammatory response of macrophages to infection [35]. Quantitative PCR was performed on a representative group of genes in the obese and lean clusters of Arg1 to determine if Arg1 was correlated with the group behavior these genes over time (Figure 4). We found that although these genes did not share the same relative expression levels as Arg1, they did share the same relative behavior as Arg1. This suggested that the SOMs should contain clustered genes based on correlation instead of expression levels. The variability of the cDNA array experiment is such that validation using RT-PCR may not translate well for all the tested time points (*i.e.* it is still possible that discrepancies exist between the two approaches) (Figure 4). However, the validation was performed to globally determine whether the leads offered by the cDNA arrays were worth pursuing. Indeed, the candidates were globally validated by RT-PCR, but most importantly confirmed by Western blot (Figure 5). Induction of Arg1 in macrophages thwarts effective immunity against intracellular pathogens due to the inhibition of macrophage M1 polarization [10], [36], consistent with our finding that a specific Arg1 inhibitor (ABH) increased the M1 polarization of BMMΦ after *P. gingivalis* infection (Figure 6).

Our results showed that the inhibition of Thbs1 and Arg1 could partially restore the M1 polarization of macrophages from obese mice exposed to live *P. gingivalis*, while having little effect in macrophages from lean mice (Figure 6). This opens up the opportunity of treatments improving the immune response to *P. gingivalis* infections that specifically target obese individuals.

The inflammatory switching genes in the macrophages from lean mice recruited pathways relating to adhesion, such as the focal adhesion and adherens junction pathway, endocytosis, and ubiquitin-mediated proteolysis (Table S2). These pathways are expected to be activated by macrophages that engulf different types of pathogens. However, the disparity of behaviors of these genes in macrophages from obese animals indicated a lack of concerted effort into activating these pathways, effectively deactivating them. In macrophages from obese mice, the switching gene Thbs1 along with its gene cluster showed an activation of proliferation and angiogenesis. The activation of these pathways may contribute to a weakened, delayed and chronic inflammation after *P. gingivalis* infection.

An apparent discrepancy is observed between the transcriptomic and proteomic levels of Thbs1 and Arg1. Both genes appear to be more expressed in lean macrophages at 4 h (Figure 4), while their corresponding proteins are preferentially detected in obese macrophages at that same time (Figure 5). A similar pattern is observed for Thbs1 in a study by Kislinger *et al.* [37]. The authors compared the mRNA and protein levels of 1,758 genes in six distinct tissues in mouse. While they concluded that there exists an overall concordance between mRNA and protein levels, several genes, including Thbs1, showed various levels of inconsistencies. Thbs1 was found to be less expressed in heart compared to placenta (2.8 fold change) and to kidney (1.7 fold change), while the Thbs1 protein was preferentially detected in heart compared to placenta (2 folds higher) and was undetected in kidney [37]. The observed discrepancies may be explained by complex post-transcriptional regulation mechanisms that are partially influenced by cell types. These mechanisms may be dysregulated in obese individuals due to an impaired metabolism. Additionally, alternative splicing may produce splice variants that have different affinities to Thbs1 cDNA probes, but that respond similarly to Thbs1 antibodies. Arg1 was not included in the study by Kislinger *et al.*, but similar explanations remain plausible.

In summary, the abnormal heightened protein production from pro-inflammatory switching genes, Thbs1 and Arg1, in macrophages from obese mice resulted in an inhibition of M1 macrophage polarization and correlated with effects on a cascade of pathways associated with proliferation and angiogenesis. These effects are potential causes of the delay in the response to infection of obese subjects. Our results suggest that therapies targeting the pro-inflammatory switching genes Thbs1 and Arg1 could increase the ability of macrophages in obese individuals, to successfully defend against *P. gingivalis* infections.

## Supporting Information

Table S1 **Primers for PCR.** Primers for the following genes were generated by Primer-BLAST (5′ to 3′): thrombospondin 1 (Thsp1), interferon regulatory factor 5 (Irf5), toll-like receptor 1 (Tlr1), toll-like receptor 7 (Tlr7), mucosa associated lymphoid tissue lymphoma translocation gene 1 (Malt1), adaptor-related protein complex 1, sigma 2 subunit (Ap1s2), paxillin (Pxn), Arginase 1 (Arg1), Janus kinase 1 (Jak1), Transforming growth factor, beta receptor II (Tgfbr2), Mammary tumor virus receptor 2 (Mtvr2), Fatty acid synthase (Fasn), Sphingomyelin phosphodiesterase, acid-like 3B (Smlpd3), and Acyloxyacyl hydrolase (Aoah).
(PDF)

Table S2 **Pathways in Lean and Obese Clusters enriched by switching genes.** Note that all enriched pathways have an EASE score <0.1 and a minimum of two genes per pathway, as per the default of DAVID. Pathways in bold are involved in cell proliferation and angiogenesis.
(PDF)
