# Supplementary material for: Partial Restoration of Macrophage Alteration from Diet-Induced Obesity in Response to Porphyromonas gingivalis Infection
Source: PLoS One. 2013 Jul 29;8(7):e70320. doi: 10.1371/journal.pone.0070320 (PMC3726386; doi:10.1371/journal.pone.0070320)
Supplement: Table S1 — Primers for PCR. Primers for the following genes were generated by Primer-BLAST (5′ to 3′): thrombospondin 1 (Thsp1), interferon regulatory factor 5 (Irf5), toll-like receptor 1 (Tlr1), toll-like receptor 7 (Tlr7), mucosa associated lymphoid tissue lymphoma translocation gene 1 (Malt1), adaptor-related protein complex 1, sigma 2 subunit (Ap1s2), paxillin (Pxn), Arginase 1 (Arg1), Janus kinase 1 (Jak1), Transforming growth factor, beta receptor II (Tgfbr2), Mammary tumor virus receptor 2 (Mtvr2), Fatty acid synthase (Fasn), Sphingomyelin phosphodiesterase, acid-like 3B (Smlpd3), and Acyloxyacyl hydrolase (Aoah). (PDF) [file pone.0070320.s001.pdf]

PCR primers

Gene	Forward Primer	Reverse Primer
Thsp1	CCATGGAGCTCCTGCGGGGA	ACGCTGGCCAGATCCCTGGT
Irf5	CAACGGCCCTGCTCCCACAG	TCCAGAGGGAGGGGCCAGGA
Tlr1	CCACATGCTGTGCCCGTCCC	CAGGCGCATGGGATTCCCCC
Tlr7	GGCCGTTGAGAGAGTTGCGGT	TTCCATGGTCCTGCTGGCCG
Malt1	GCTGCCGGGCAACTGGACAT	TGCCTGGCACCAGCCTTTGG
Ap1s2	CTTCCACAGCACGGCGACGG	GCAGGAGATCGGCCTGCTCA
Pxn	GGGCAGGACGAGGGAGGGTT	TAGGGCTGTCCGTCCCGCTC
Arg1	ACCACGGGGACCTGGCCTTT	CCTGGCGTGGCCAGAGATGC
Jak1	GCTGCGGCAGGAAGGGAGTG	AGGCTGCCACTCCTGGGCTT
Tgfbr2	ACGTTCCCAAGTCGGATGTGGA	GCTCTCCCAGGACGGGCTCA
Mtvr2	TGTTGTGCCAGCTGTGGGGC	GCAACCACTGGTCACGGGCA
Fasn	ACGACAGCAACCTCACGGCG	CAGCCAGCACCTCTGCCACC
Smlpd3	GAGGCCTACCAGGTGCCGGA	AGTCAGTGGGTGGGCTGGCA
Aoah	TACCACTGGCGAGGGCGTGA	CATCGGTAAGGGCGGACGGC
