# Supplementary material for: Partial Restoration of Macrophage Alteration from Diet-Induced Obesity in Response to Porphyromonas gingivalis Infection
Source: PLoS One. 2013 Jul 29;8(7):e70320. doi: 10.1371/journal.pone.0070320 (PMC3726386; doi:10.1371/journal.pone.0070320)
Supplement: Table S2 — Pathways in Lean and Obese Clusters enriched by switching genes. Note that all enriched pathways have an EASE score <0.1 and a minimum of two genes per pathway, as per the default of DAVID. Pathways in bold are involved in cell proliferation and angiogenesis. (PDF) [file pone.0070320.s002.pdf]

Switching Gene	Lean	Obese
Thrombospondin 1	Focal Adhesion<br>**Toll-Like Receptor Signaling Pathway**<br>Adherens Junction	Focal Adhesion<br>Regulation of Actin<br>**Bladder Cancer**
Arginase 1	Ubiquitin Mediated Proteolysis<br>Endocytosis<br>Chronic Myeloid Leukemia<br>**Pathways in Cancer**	Arginine and Proline Metabolism
Chemokine (C-C motif) receptor 5	O-Glycan Biosynthesis	MAPK Signaling Pathway<br>JAK-STAT Signaling Pathway
Histocompatibility 2, Q region locus 6	Cytosolic DNA-sensing Pathway<br>Glycine, Serine and Threonine Metabolism	Axon Guidance<br>Ubiquitin Mediated Proteolysis
Collagen, type III, alpha 1	JAK-STAT Signaling Pathway<br>ECM Receptor Interaction<br>MAPK Signaling Pathway	Lysosome<br>Endocytosis<br>Phosphatidylinositol Signaling System<br>Regulation of Actin Cytoskeleton<br>Focal Adhesion<br>**Glioma**<br>Oocyte Meiosis<br>**VEGF Signaling Pathway**
Caldesmon 1	Regulation of Actin Cytoskeleton<br>Focal Adhesion<br>Vascular Smooth Muscle Contraction<br>B-cell Receptor Signaling Pathway	Spliceosome<br>Ubiquitin Mediated Proteolysis<br>**TGF-beta Signaling Pathway**<br>Endocytosis<br>Renal Cell Carcinoma<br>Chronic Myeloid Leukemia<br>Cell Cycle<br>Neurotrophin Signaling Pathway
Collagen, type I, alpha 2,2	Regulation of Actin Cytoskeleton<br>**Glioma**<br>Melanoma<br>Chronic Myeloid Leukemia<br>Oocyte Meiosis<br>B-cell Receptor Signaling Pathway<br>Focal Adhesion<br>Hematopoietic Cell Lineage<br>Cell Cycle<br>**Prostate Cancer**<br>Neurotrophin Signaling Pathway<br>Ubiquitin Mediated Proteolysis<br>Fc gamma R-mediated Phagocytosis	Oxidative Phosphorylation<br>Alzheimer's Disease<br>Huntington's Disease<br>Parkinson's Disease<br>Oocyte Meiosis<br>Leukocyte Transendothelial Migration<br>Citrate Cycle (TCA cycle)<br>Neurotrophin Signaling Pathway<br>Glyoxylate and Dicarboxylate Metabolism<br>Lysosome<br>**Endometrial Cancer**<br>Cell Cycle<br>Insulin Signaling Pathway<br>**Thyroid Cancer**<br>Melanogenesis<br>**Pathways in Cancer**
Collagen, type V, alpha 2	MAPK Signaling Pathway<br>ErbB Signaling Pathway<br>Ubiquitin Mediated Proteolysis<br>Focal Adhesion<br>Regulation of Actin Cytoskeleton	Prostate Cancer<br>Focal Adhesion<br>Endocytosis<br>Inositol Phosphate Metabolism<br>Fc gamma R-mediated Phagocytosis<br>JAK-STAT Signaling Pathway<br>Phosphatidylinositol Signaling System<br>T-cell Receptor Signaling Pathway<br>Natural Killer Cell Mediated Cytotoxicity<br>B-cell Receptor Signaling Pathway<br>Progesterone-mediated Oocyte Maturatoin<br>Apoptosis<br>**Chemokine Signaling Pathway**<br>Ubiquitin Mediated Proteolysis<br>**Endometrial Cancer**<br>Insulin Signaling Pathway<br>Acute Myeloid Leukemia<br>Regulation of Actin Cytoskeleton<br>RIG-I-like Receptor Signaling Pathway<br>**Renal Cell Carcinoma**<br>**Pancreatic Cancer**<br>Chronic Myeloid Leukemia<br>Cell Cycle<br>Neurotrophin Signaling Pathway<br>**Small Cell Lung Cancer**<br>**Colorectal Cancer**<br>ErbB Signaling Pathway<br>**Pathways in Cancer**
Biglycan	Vascular Smooth Muscle Contraction<br>Spliceosome<br>Axon Guidance<br>Endocytosis<br>Insulin Signaling Pathway<br>Gap Junction<br>Apoptosis<br>Chemokine Signaling Pathway<br>MAPK Signaling Pathway<br>Acute Myeloid Leukemia	Citrate Cycle (TCA cycle)<br>Huntington's Disease<br>Amino Sugar and Nucleotide Sugar Metabolism<br>Oxidative Phosphorylation<br>Parkinson's Disease<br>O-Glycan Biosynthesis<br>Prion Diseases<br>Alzheimer's Disease
